# Characteristics and outcomes of hospitalised patients with acute kidney injury and COVID-19

**DOI:** 10.1371/journal.pone.0241544

**Published:** 2020-11-03

**Authors:** Patrick Hamilton, Prasanna Hanumapura, Laveena Castelino, Robert Henney, Kathrine Parker, Mukesh Kumar, Michelle Murphy, Tamer Al-Sayed, Sarah Pinnington, Tim Felton, Rachael Challiner, Leonard Ebah

**Affiliations:** 1 Manchester Institute of Nephrology and Transplantation, Manchester University NHS Foundation Trust, Manchester, United Kingdom; 2 Manchester Academic Health Sciences Centre (MAHSC), Manchester, United Kingdom; 3 Division of Cell Matrix Biology and Regenerative Medicine, Wellcome Centre for Cell-Matrix Research, School of Biological Sciences, Faculty of Biology Medicine and Health, The University of Manchester, Manchester, United Kingdom; 4 Division of Infection, Immunity and Respiratory Medicine, Faculty of Biology, Medicine and Health, The University of Manchester, Manchester, United Kingdom; 5 Intensive Care Unit, Wythenshawe Hospital, Manchester University NHS Foundation Trust, Manchester, United Kingdom; 6 NIHR Manchester Biomedical Research Centre, Manchester, United Kingdom; Erasmus Medical Center, NETHERLANDS

## Abstract

**Introduction:**

COVID-19 has spread globally to now be considered a pandemic by the World Health Organisation. Initially patients appeared to have a respiratory limited disease but there are now increasing reports of multiple organ involvement including renal disease in association with COVID-19. We studied the development and outcomes of acute kidney injury (AKI) in patients with COVID-19, in a large multicultural city hospital trust in the UK, to better understand the role renal disease has in the disease process.

**Methods:**

This was a retrospective review using electronic records and laboratory data of adult patients admitted to the four Manchester University Foundation Trust Hospitals between March 10 and April 30 2020 with a diagnosis of COVID-19. Records were reviewed for baseline characteristics, medications, comorbidities, social deprivation index, observations, biochemistry and outcomes including mortality, admission to critical care, mechanical ventilation and the need for renal replacement therapy.

**Results:**

There were 1032 patients included in the study of whom 210 (20.3%) had AKI in association with the diagnosis of COVID-19. The overall mortality with AKI was considerably higher at 52.4% compared to 26.3% without AKI (p-value <0.001). More patients with AKI required escalation to critical care (34.8% vs 11.2%, p-value <0.001). Following admission to critical care those with AKI were more likely to die (54.8% vs 25.0%, p-value <0.001) and more likely to require mechanical ventilation (86.3% vs 66.3%, p-value 0.006).

**Discussion:**

We have shown that the development of AKI is associated with dramatically worse outcomes for patients, in both mortality and the requirement for critical care. Patients with COVID-19 presenting with, or at risk of AKI should be closely monitored and appropriately managed to prevent any decline in renal function, given the significant risk of deterioration and death.

## Introduction

In December 2019, the first cases of a cluster of pneumonia of unknown cause was described in Wuhan, China, later identified as a novel coronavirus—SARS Coronavirus 2 (SARS-Cov-2) [1, 2]. The clinical disease, later called COVID-19, resulted from the virus spreading globally during the first few months of 2020 to be declared a global pandemic on the 11th March 2020 [3]. The clinical course has a predominant respiratory presentation leading to acute respiratory failure requiring ventilatory support and intensive care supportive treatment [4–7]. Early reports from Wuhan showed very little evidence of Acute Kidney Injury (AKI) in patients with COVID-19. In February 2020, Wang et al. reported no episodes of AKI in 116 hospitalized patients with COVID-19 in Wuhan, China [8]. Conversely, in a retrospective analysis of 333 patients admitted with COVID-19 pneumonia, Pei at al found renal involvement (either AKI or urine dipstick analysis or both) in 75.4% of the patients with Covid-19 [9]. Patients with Covid-19 who developed AKI had a near 10-fold higher mortality (11.2%) compared to those without AKI (1.2%).

Several further studies have focused attention on AKI in critically and non-critically ill patients with COVID-19. In March 2020, Cheng at al reported on a prospective cohort of 701 COVID-19 cases in Tongji Hospital, Wuhan, China and found that 43.9% of these patients had proteinuria during hospitalisation with AKI occurring in 5.1% [10]. AKI stage 2 (OR 3.51,1.49–8.26), and stage 3 (OR 4.38, 2.31–8.31) were independent risk factors for increased in-hospital mortality in an adjusted Cox proportional hazard model. This has brought more attention to the potential hazards of AKI in COVID-19 disease including understanding the epidemiology, pathophysiology, and ultimately prevention, treatment and reducing its impact. A renal histopathological analysis of 26 patients post mortem showed evidence of direct invasion of the renal tissue by SARS-Cov-2 [11]. Using available evidence, a multifactorial AKI pathogenesis model including hypovolaemia, cytokine storm, endothelial dysfunction, ARDS, complement activation, angiotensin II overactivity, direct viral attack of podocyte and tubules, myocardial dysfunction, and hypercoagulability has been proposed [12, 13].

As the incidence of COVID-19 rose across European countries and the USA, reports showed that AKI was a more predominant feature of the condition than seen in initial reports from China. In April 2020, Richardson et al. described the presenting characteristics, co-morbidities and outcomes of 5700 patients admitted with COVID-19 to New York Hospitals in the USA, and of the 2626 discharged dead or alive [14]. This paper showed a much higher than previously reported incidence of AKI with COVID-19, as 523 patients in their whole cohort (22.2%), and a third of their patients over 65 years developed AKI (as defined by Kidney Disease Improving Global outcomes (KDIGO) [15]. A little later, in May 2020, Hirsch et al., reported even higher rates of AKI; 36.6% overall in 1993 out of 5449 patients admitted with COVID-19 to 13 New York Metropolitan Area hospitals [16]. The incidence of AKI stage 3 was 31.1%, and 14.3% of all AKI patients required renal replacement therapy (RRT). There seemed a temporal association between the requirement for mechanical ventilation and AKI, as half of those requiring mechanical ventilation developed AKI within 24 hours. At the time of reporting, 35% of the patients with AKI had died. Wilbers and Koning described a 60% incidence and 41% mortality of AKI among a cohort of 76 Critically ill patients with COVID-19 in the Netherlands, rather similar to the findings of Fominskiy et al. in Milan, Italy [17, 18].

The COVID-19 pandemic extended to the UK in March 2020, with peak infection rates seen between early and mid-April 2020 [19]. Our Hospital Trust in Manchester (UK’s third largest city) is one of the largest secondary and tertiary care Trusts in the UK’s National Health Service, with over 2000 inpatient beds. Acute Kidney Injury has previously been reported by our trust, and other authors, to have a high incidence in hospitalised patients, with worse outcomes than in those without AKI [20, 21]. Our Hospital Trust has already implemented a successful comprehensive quality improvement programme to improve AKI care and outcomes, and has a consistent and continuing fall in AKI incidence, time to recovery, and length of stay [22].

At the height of the COVID-19 pandemic locally, the trust admitted cumulatively over 1000 patients between March and May 2020. This paper sets out to further understand the epidemiology, clinical characteristics and outcomes of hospitalized patients with COVID-19 disease who developed AKI. To our knowledge, this study is the largest cohort of hospitalized COVID-19 and AKI patients, including critically ill patients in ICU stetting’s, and ward-based patients in United Kingdom or Europe.

## Methods

This was a retrospective review using electronic records and laboratory data of adult (age >18 years) patients admitted to Manchester University Foundation Trust Hospitals between March 1 and April 30 2020 and had a confirmed clinical and/or virologic diagnosis of COVID-19. After consultation with the National Health Service Research Ethics Committee (NHS REC) and the Trust Research and Development department (Manchester University Hospitals NHS Foundation Trust), the study was exempt from requiring a specific ethical approval and therefore waived, as it was considered a retrospective audit and considered a service evaluation. Given this, no consent was required or obtained. Data for this study was obtained from four different hospitals using the electronic health records such as Patient administration service (PAS) for hospital admissions and co-morbidities, ICE and Sunquest ICE for laboratory data and Patientrack™ by Alcidion for observations data. All data was manually checked to exclude duplicate values. All patients’ information and data was anonymised for analysis and reporting.

### Setting

Manchester University NHS Foundation Trust (MFT) Hospitals is the leading provider of tertiary and specialist healthcare services in Greater Manchester. It comprises 10 hospitals/managed clinical services across six separate sites, providing a wide range of services from comprehensive local general hospital care through to highly specialised regional and national services. The trust is the main provider of hospital care to approximately 750,000 people in Manchester and Trafford and the single biggest provider of specialised services in the North West of England. It is also the lead provider for a significant number of specialised services including Breast Care, Vascular, Cardiac, Renal, Renal Transplant Respiratory, Urology, Paediatrics, Women’s Services, Ophthalmology and Genomic Medicine. It is also the main teaching hospital affiliated to the University of Manchester.

### Study design and cohort

Data for this study was obtained from four of the trust’s hospitals that cared for adult inpatients with COVID-19 infection (Manchester Royal Infirmary, Wythenshawe Hospital, Saint Mary’s Hospital and Trafford General Hospital). All adult patients (age ≥ 18 years), including the current inpatients and any new admissions from 10th of March to 30th of April 2020 (51 days), who tested positive by polymerase chain reaction testing of a nasopharyngeal or lower respiratory tract sample for COVID-19, were eligible. Patients who were transferred between hospitals within our own trust were treated as one hospital episode and the first admission nearest to the COVID 19 result date was considered. Day cases and all known Haemodialysis patients were excluded. Records were abstracted for demographic and co-morbidity data. Postal area codes were used to link to government calculated Deprivation Indices [23]. The patients were followed up for 30 days from the date of becoming identified as COVID positive, and in patients where the COVID positive date was before admission, then 30 days from admission for all outcome data. Day 0 taken as the date of COVID-19 positive result.

### Definitions and measurement

#### Acute kidney injury (AKI)

Once the cohort was identified, laboratory data was matched for confirmation of AKI defined according to Kidney Disease: Improving Global Outcomes (KDIGO) [15] criteria as follows: stage 1—increase in serum creatinine by 26.5micromol/L within 48 hours or a 1.5 to 1.9 times increase in serum creatinine from baseline within 7 days; stage 2–2.9 times increase in serum creatinine within 7 days; stage 3–3 times or more increase in serum creatinine within seven days or initiation of RRT. Although an electronic AKI detection and alerting system has already been in place within the trust since March 2016 (Think Kidneys/NPSA Safety Alert NHS/PSA/RE/2016/007) manual confirmation of each AKI case was still carried out, to ensure a valid baseline creatinine result was utilised. All in- patients who had AKI from seven days prior to COVID positive date and any date after the positive result date were included. Any in-patients who had developed AKI and recovered prior to seven days before the COVID positive date were excluded. Urine output criteria to define AKI were not utilised, as the documentation of urine output was deemed unreliable. Any patient requiring renal replacement therapy (RRT), regardless of AKI stage, was defined as stage 3 AKI, as per KDIGO criteria.

#### Blood results

Blood results for all the patients were collected from data collectable tables derived from the trusts clinical laboratory reporting system (ICE® and SUNQEST ICE®). Two sets of blood results data were analysed and defined as baseline and peak results. Baseline results were the results on day 0 (day of the first positive COVID sample) and if no blood tests on that day then the sample nearest to the day 0 result (no greater than 7 days earlier were used). The second set of blood test results (peak results) were taken up to 30 days from the COVID positive date, where the highest and lowest ranges of the selected blood results were identified. If there is no repeat sample blood test, then these patients were excluded for the peak blood results analysis.

#### Medications

Review of medication history was undertaken to check if the cohort patient was prescribed an angiotensin-converting enzyme (ACE) inhibitor and/or an angiotensin receptor blocker (ARB). This was collected manually by reviewing discharge summaries and General Practitioner records.

#### Physiological observations

Patientrack™ by Alcidion is an electronic clinical observation system which is used to collect the observations at the bedside of patients across MFT. Observation data (temperature, blood pressure, mean arterial pressure (MAP), respiratory rate, oxygen saturations and early warning score (EWS)) for the patient cohort was collected for 72 hours around the time of COVID diagnosis (24 hrs before COVID positive date, 24 hrs day of COVID positive date and 24 hrs after COVID positive date). The nearest set of observations to COVID-19 positive date were considered.

#### Comorbidities

Comorbidities based on ICD-10 codes obtained from patient electronic records as described above. Definitions of comorbidities and Charlson comorbidity score calculated as described by Quan et al. and Charlson et al. respectively, and using R Comorbidity package [24–26].

### Data availability

The Raw data required to reproduce these findings cannot be shared due to patient privacy, legal and ethical reasons. However, the processed data that support the findings of this study are available from the corresponding author upon reasonable request.

### Statistical analysis

Categorical variables presented as number and percentage. Normality assessed using quantile-quantile (QQ) plots and Shapiro-Wilk normality test. Non-parametric continuous variables presented as median and interquartile range. Risk factors impacting on the development of AKI was assessed with the use of logistic regression. Multivariable models adjusted for demographics and variables showing significance at univariable analysis. Model 1 included vital signs as the amalgamated Early Warning Score [27] (EWS) at diagnosis of COVID-19. Model 2 included vital signs individually and without the EWS. Survival analysis carried out using Kaplan-Meier method and Cox proportional regression model. Multivariable model adjusted for demographics and variables showing significance at univariable analysis. Significance taken as p-value less than 0.05. All analysis carried out using R version 3.6.1 [28].

## Results

### Demographics

From March 10, 2020 to April 30, 2020, 11,721 patients were admitted to the four hospitals, and 1166 of these had a diagnosis of COVID-19. After excluding the 35 dialysis patients and 99 day-cases, there were a total of 1032 COVID-19 positive patients included in the analysis; 20.3% (n = 210) of these had a diagnosis of AKI, and 79.7% (n = 822) patients never developed AKI. The median age at diagnosis for the overall cohort was 71 years (IQR 56–83), with 55.1% (n = 569) males and 44.9% (n = 463) females. Table 1 for full demographics.

**Table 1:** Demographics table.

			AKI		
	level	Total population	No	Yes	p-value
n		1032	822	210	
Age	*Median (IQR)*	71 (56–83)	71 (56–83)	71.5 (58–81)	0.826
Sex (%)	*Female*	463 (44.9)	384 (46.7)	79 (37.6)	0.022
	*Male*	569 (55.1)	438 (53.3)	131 (62.4)	
Ethnicity (%)	*White*	725 (70.3)	586 (71.3)	139 (66.5)	0.012
	*Asian*	116 (11.3)	95 (11.6)	21 (10.0)	
	*Black*	94 (9.1)	63 (7.7)	31 (14.8)	
	*Mixed*	14 (1.4)	12 (1.5)	2 (1.0)	
	*Other*	28 (2.7)	26 (3.2)	2 (1.0)	
	*Unknown*	54 (5.2)	40 (4.9)	14 (6.7)	
	*NA*	1 (0.1)	0 (0.0)	1 (0.5)	
RAASi (%)	*No*	232 (22.5)	170 (20.7)	62 (29.5)	0.008
IMD Decile	*1*	283 (27.5)	235 (28.7)	48 (22.9)	0.565
	*2*	118 (11.5)	91 (11.1)	27 (12.9)	
	*3*	112 (10.9)	84 (10.3)	28 (13.3)	
	*4*	106 (10.3)	79 (9.7)	27 (12.9)	
	*5*	58 (5.6)	50 (6.1)	8 (3.8)	
	*6*	50 (4.9)	40 (4.9)	10 (4.8)	
	*7*	77 (7.5)	61 (7.5)	16 (7.6)	
	*8*	97 (9.4)	78 (9.5)	19 (9.0)	
	*9*	44 (4.3)	36 (4.4)	8 (3.8)	
	*10*	83 (8.1)	64 (7.8)	19 (9.0)	
	*NA*	4 (0.4)	4 (0.5)	0 (0.0)	
CCI Score	*Median (IQR)*	1.41 (1.28)	1.37 (1.28)	1.58 (1.27)	0.047
Myocardial Infarction	*No*	851 (82.5)	690 (83.9)	161 (76.7)	0.003
	*Yes*	83 (8.0)	67 (8.2)	16 (7.6)	
	*NA*	98 (9.5)	65 (7.9)	33 (15.7)	
Congestive Heart Failure	*No*	805 (78.0)	651 (79.2)	154 (73.3)	0.003
	*Yes*	129 (12.5)	106 (12.9)	23 (11.0)	
	*NA*	98 (9.5)	65 (7.9)	33 (15.7)	
Peripheral Vascular Disease	*No*	870 (84.3)	711 (86.5)	159 (75.7)	<0.001
	*Yes*	64 (6.2)	46 (5.6)	18 (8.6)	
	*NA*	98 (9.5)	65 (7.9)	33 (15.7)	
Cerebrovascular disease	*No*	889 (86.1)	723 (88.0)	166 (79.0)	0.002
	*Yes*	45 (4.4)	34 (4.1)	11 (5.2)	
	*NA*	98 (9.5)	65 (7.9)	33 (15.7)	
Dementia	*No*	777 (75.3)	631 (76.8)	146 (69.5)	0.003
	*Yes*	157 (15.2)	126 (15.3)	31 (14.8)	
	*NA*	98 (9.5)	65 (7.9)	33 (15.7)	
COPD	*No*	675 (65.4)	543 (66.1)	132 (62.9)	0.002
	*Yes*	259 (25.1)	214 (26.0)	45 (21.4)	
	*NA*	98 (9.5)	65 (7.9)	33 (15.7)	
Rheumatoid Disease	*No*	911 (88.3)	737 (89.7)	174 (82.9)	0.002
	*Yes*	23 (2.2)	20 (2.4)	3 (1.4)	
	*NA*	98 (9.5)	65 (7.9)	33 (15.7)	
Peptic Ulcer Disease	*No*	925 (89.6)	749 (91.1)	176 (83.8)	0.002
	*Yes*	9 (0.9)	8 (1.0)	1 (0.5)	
	*NA*	98 (9.5)	65 (7.9)	33 (15.7)	
Mild Liver Disease	*No*	904 (87.6)	729 (88.7)	175 (83.3)	0.001
	*Yes*	30 (2.9)	28 (3.4)	2 (1.0)	
	*NA*	98 (9.5)	65 (7.9)	33 (15.7)	
Diabetes without complications	*No*	697 (67.5)	564 (68.6)	133 (63.3)	0.003
	*Yes*	237 (23.0)	193 (23.5)	44 (21.0)	
	*NA*	98 (9.5)	65 (7.9)	33 (15.7)	
Diabetes with complications	*No*	898 (87.0)	736 (89.5)	162 (77.1)	<0.001
	*Yes*	36 (3.5)	21 (2.6)	15 (7.1)	
	*NA*	98 (9.5)	65 (7.9)	33 (15.7)	
Hemi or Paraplegia	*No*	917 (88.9)	742 (90.3)	175 (83.3)	0.002
	*Yes*	17 (1.6)	15 (1.8)	2 (1.0)	
	*NA*	98 (9.5)	65 (7.9)	33 (15.7)	
Renal Disease	*No*	790 (76.6)	659 (80.2)	131 (62.4)	<0.001
	*Yes*	144 (14.0)	98 (11.9)	46 (21.9)	
	*NA*	98 (9.5)	65 (7.9)	33 (15.7)	
Mod/Severe Liver Disease	*No*	922 (89.3)	748 (91.0)	174 (82.9)	0.002
	*Yes*	12 (1.2)	9 (1.1)	3 (1.4)	
	*NA*	98 (9.5)	65 (7.9)	33 (15.7)	
Cancer	*No*	862 (83.5)	705 (85.8)	157 (74.8)	<0.001
	*Yes*	72 (7.0)	52 (6.3)	20 (9.5)	
	*NA*	98 (9.5)	65 (7.9)	33 (15.7)	
Metastatic cancer	*No*	910 (88.2)	739 (89.9)	171 (81.4)	0.002
	*Yes*	24 (2.3)	18 (2.2)	6 (2.9)	
	*NA*	98 (9.5)	65 (7.9)	33 (15.7)	
AIDS	*No*	933 (90.4)	756 (92.0)	177 (84.3)	0.002
	*Yes*	1 (0.1)	1 (0.1)	0 (0.0)	
	*NA*	98 (9.5)	65 (7.9)	33 (15.7)	

RAASi–Renin-angiotensin-aldosterone-system inhibitors; IMD–Index of multiple deprivation; CCI–Charlson Comorbidity index; COPD–Chronic Obstructive Pulmonary Disease.

There were significant differences between the ethnicity of the two cohorts although the majority of patients admitted were White British, with 71.3% (n = 586) in the non-AKI cohort and 66.5% (n = 139) in the AKI cohort. There were more Black patients in the AKI cohort with 14.8% (n = 31), compared to the non-AKI cohort with 7.7% (n = 63); p-value 0.012. There were also significantly more males in the AKI group compared to the non-AKI group, p-value 0.022.

There were more patients on RAAS inhibition in the AKI group with 29.5% (n = 62), compared to the non-AKI group with 20.7% (n = 170; p = 0.008). Patients in the AKI groups were more likely to have had a previous diagnosis of renal disease compared to those in the non-AKI group (p-value <0.001). Diabetes with complications was significantly higher in the AKI cohort with 8.5% (n = 15) patients compared to 2.8% (n = 21) in the non-AKI cohort, p-value 0.001, but no difference between the two groups in patients with diabetes without complications (p-value 0.937).

### Incidence and severity of AKI

AKI occurred in 210 patients (20.3%). At the time of initial diagnosis of their AKI then 58% (n = 123) were in stage 1, 18% (n = 37) stage 2 and 24% (n = 50) were in stage 3 AKI At peak AKI stage then 36% (n = 75) had stage 1, 20% (n = 43) stage 2 and 44% (n = 92) had stage 3 AKI. AKI worsened in 31% (n = 65) of the patients. 59% (n = 123) of patients had admission AKI and 42% (n = 87) patients developed AKI post admission. The median time to the initial AKI from the date of COVID-19 positivity was -1.00 days (IQR -1.00–2.00). 52% (n = 110) of patients were in AKI during the 72 hours of COVID-19 positive date (i.e. 24hrs before, 24 hours of positive date and 24hrs post COVID-19) and 24% (n = 50) of patients developed AKI post 24hrs of COVID-19 positive date.

The total number of patients requiring renal replacement therapy (RRT) was 32 (15.2%), and this was provided for 24 patients by continuous RRT alone, seven patients receiving both continuous RRT and haemodialysis and one patient needing haemodialysis only.

Of the 32 patients needing RRT 65.6% (n = 21) died, two patients needed long term haemodialysis and the rest recovering their kidney function (n = 8).

### Baseline blood results

At baseline, patients with a higher serum Sodium (OR 1.04, 95% CI 1.02–1.06, p<0.001), Urea (OR 1.16, 95% CI 1.13–1.19, p<0.001) and Creatinine (OR 1.01, 95% CI 1.01–1.01, p<0.001) were more likely to have AKI compared to those in the non-AKI cohort. There was no statistical difference in the lymphocyte titre between the groups (p-value 0.718) and it did not appear to be a risk factor for developing AKI; OR 1.02, 95% CI 0.88–1.16, p = 0.719. Patients in the AKI cohort had a higher baseline CRP, with a median of 104.00nmol/L (IQR 51.00–196.00) compared to 59.00nmol/L (IQR 23.00–119.25), and was a significant risk factor for the development of AKI; OR 1.01, 95% CI 1.00–1.01, p<0.001. Patients with a previous diagnosis of Renal disease were more likely to develop AKI (OR 2.36, 95% CI 1.58–3.50, p-value < 0.001), as were those with Diabetes with complications (OR 3.25, 95% CI 1.61–6.39, p-value 0.001). Tables 2 and 3.

**Table 2:** Results table. Categorical variables presented as n (%) and continuous as median (IQR).

			AKI		
		Overall	No	Yes	P-value
n		1032	822	210	
Died		326 (31.6)	216 (26.3)	110 (52.4)	<0.001
30-day mortality–number who died		278 (26.9)	181 (22.0)	97 (46.2)	<0.001
Readmitted within 30 days		86 (8.3)	73 (8.9)	13 (6.2)	0.263
Required RRT		32 (3.1)	0 (0.0)	32 (15.2)	<0.001
Length of stay (days)		10.00 (4.00–25.00)	9.00 (4.00–23.00)	16.00 (7.25–35.75)	<0.001
Length of stay from Day Zero (days)		8.00 (4.00–19.00)	7.00 (4.00–17.00)	13.00 (6.25–27.75)	<0.001
Time from COVID positivity to AKI (days)		-1.00 (-1.00–2.00)	NA (NA-NA)	-1.00 (-1.00–2.00)	NA
Day Zero	*Sodium (mmol/L)*	137.00 (134.00–141.00)	137.00 (135.00–140.00)	138.00 (133.00–142.00)	0.568
	*Urea (mmol/L)*	6.70 (4.60–10.30)	6.00 (4.30–8.70)	11.20 (7.50–17.30)	<0.001
	*Creatinine (μmol/L)*	82.00 (64.00–114.00)	78.00 (61.00–99.00)	131.00 (94.00–217.00)	<0.001
	*Haemoglobin (g/L)*	124.00 (106.00–141.00)	125.00 (108.00–141.00)	120.00 (103.00–142.00)	0.101
	*CRP (nmol/L)*	68.00 (27.00–132.00)	59.00 (23.00–119.25)	104.00 (51.00–196.00)	<0.001
	*Lymphocytes (10*^*9*^*/L)*	0.94 (0.65–1.31)	0.95 (0.66–1.36)	0.91 (0.62–1.24)	0.100
Peak	*Sodium (mmol/L)*	141.00 (138.00–145.00)	140.00 (137.00–143.00)	145.00 (140.00–153.00)	<0.001
	*Urea (mmol/L)*	8.30 (5.40–13.70)	7.00 (4.90–10.35)	18.40 (12.80–27.30)	<0.001
	*Creatinine (μmol/L)*	91.00 (69.00–131.00)	81.00 (65.00–104.00)	189.00 (136.00–305.00)	<0.001
	*CRP (nmol/L)*	115.00 (48.25–215.00)	98.00 (40.00–184.00)	208.00 (107.00–312.00)	<0.001
Minimum	*Haemoglobin (g/L)*	112.00 (94.00–128.00)	114.00 (97.00–130.00)	96.00 (76.00–117.00)	<0.001
	*Lymphocyte (10*^*9*^*/L)*	0.76 (0.51–1.10)	0.80 (0.53–1.17)	0.64 (0.41–0.89)	<0.001
Early Warning Score (EWS)		1.00 (0.00–2.00)	1.00 (0.00–2.00)	1.00 (0.00–3.00)	0.006
Temperature (Celsius)		36.60 (36.30–37.10)	36.60 (36.30–37.10)	36.50 (36.20–37.00)	0.026
Systolic blood pressure (mmHg)		126.00 (111.00–142.00)	127.00 (112.00–142.00)	124.00 (108.00–142.00)	0.253
Diastolic blood pressure (mmHg)		71.00 (62.25–81.00)	71.00 (63.00–81.00)	70.00 (62.00–80.00)	0.282
Mean Arterial Pressure (mmHg)		90.00 (80.00–99.00)	91.00 (80.00–100.00)	89.00 (78.00–99.00)	0.268
Oxygen Saturations (Percent)		96.00 (94.00–97.00)	96.00 (94.00–97.00)	95.00 (94.00–97.00)	0.127
Respiratory Rate (breaths per minute)		19.00 (17.00–22.00)	19.00 (17.00–22.00)	20.00 (18.00–24.00)	0.026
Went to Critical Care		165 (16.0)	92 (11.2)	73 (34.8)	<0.001
Died following admission to Critical Care		63 (38.2)	23 (25.0)	40 (54.8)	<0.001
Died in Critical Care		59 (35.8)	21 (22.8)	38 (52.1)	<0.001
Required mechanical ventilation		124 (75.2)	61 (66.3)	63 (86.3)	0.006

RRT–renal replacement therapy; CRP–C-Reactive protein.

**Table 3:** Logistic regression results for development of AKI. Multivariable models adjusted by demographics and variables showing significance at univariable analysis. Multivariable model 1: vital signs included as EWS, and not individually. Multivariable Model 2: vital signs included as separate variables, and not including EWS.

		OR (Univariable)	OR (Multivariable 1)	OR (Multivariable 2)
Age		1.00 (0.99–1.01, p = 0.439)	1.01 (0.99–1.02, p = 0.389)	1.01 (0.99–1.02, p = 0.480)
Sex	*Female*			
	*Male*	1.45 (1.07–1.99, p = 0.018)	1.59 (1.07–2.38, p = 0.024)	1.61 (1.08–2.42, p = 0.021)
Ethnicity	*White*			
	*Asian*	0.93 (0.55–1.52, p = 0.785)	0.69 (0.32–1.41, p = 0.334)	0.78 (0.36–1.57, p = 0.500)
	*Black*	2.07 (1.29–3.29, p = 0.002)	1.31 (0.69–2.39, p = 0.397)	1.38 (0.72–2.54, p = 0.319)
	*Mixed*	0.70 (0.11–2.61, p = 0.647)	0.52 (0.03–3.36, p = 0.559)	0.57 (0.03–3.84, p = 0.626)
	*Other*	0.32 (0.05–1.10, p = 0.128)	0.30 (0.02–1.61, p = 0.262)	0.28 (0.01–1.50, p = 0.229)
	*Unknown*	1.48 (0.76–2.73, p = 0.231)	1.16 (0.44–2.74, p = 0.750)	1.13 (0.42–2.72, p = 0.793)
BAME	*White*			
	*BAME*	1.20 (0.84–1.70, p = 0.297)		
	*Unknown*	1.48 (0.76–2.73, p = 0.231)		
RAASi	*Yes*	1.61 (1.14–2.25, p = 0.006)	1.55 (1.00–2.37, p = 0.046)	1.50 (0.96–2.30, p = 0.068)
IMD Decile		1.01 (0.96–1.06, p = 0.629)		
Diabetes w/ comp.		3.25 (1.61–6.39, p = 0.001)	2.29 (0.94–5.48, p = 0.063)	2.38 (0.96–5.74, p = 0.055)
Renal disease		2.36 (1.58–3.50, p<0.001)	2.43 (1.50–3.92, p<0.001)	2.30 (1.41–3.73, p = 0.001)
Day zero	*Sodium (mmol/L)*	1.04 (1.02–1.06, p<0.001)	1.04 (1.01–1.07, p = 0.014)	1.04 (1.01–1.07, p = 0.018)
	*Urea (mmol/L)*	1.16 (1.13–1.19, p<0.001)		
	*Creatinine*	1.01 (1.01–1.01, p<0.001)		
	*Haemoglobin*	0.99 (0.99–1.00, p = 0.090)		
	*Lymphocyte*	1.02 (0.88–1.16, p = 0.719)		
	*CRP*	1.01 (1.00–1.01, p<0.001)	1.00 (1.00–1.01, p = 0.002)	1.00 (1.00–1.01, p = 0.002)
EWS		1.18 (1.09–1.28, p<0.001)	1.11 (1.00–1.23, p = 0.044)	
Temperature	*Celsius*	0.78 (0.62–0.97, p = 0.030)		0.83 (0.63–1.07, p = 0.155)
Blood pressure	*Systolic (mmHg)*	1.00 (0.99–1.00, p = 0.317)		1.08 (0.85–1.37, p = 0.544)
	*Diastolic (mmHg)*	1.00 (0.98–1.01, p = 0.488)		1.17 (0.72–1.89, p = 0.528)
MAP	*mmHg*	0.99 (0.98–1.01, p = 0.347)		0.79 (0.38–1.62, p = 0.518)
Oxygen Saturations	*Percent*	0.94 (0.90–0.98, p = 0.005)		0.98 (0.93–1.03, p = 0.503)
Respiratory rate	*Breaths/min*	1.04 (1.02–1.07, p = 0.002)		1.03 (0.99–1.06, p = 0.128)

BAME–Black, Asian and Minority Ethnic; RAASi–Renin-angiotensin-aldosterone-system inhibitors; IMD–Index of multiple deprivation; Diabetes w/ comp.–Diabetes with complications; CCI–Charlson Comorbidity index; EWS–early warning score; MAP–mean arterial pressure.

Multiple logistic regression analysis shows that baseline serum sodium (OR 1.04, 95% CI 1.01–1.07, p = 0.014), CRP (OR 1.00, 95% CI 1.00–1.01, p = 0.002), male sex (OR 1.59, 95% CI 1.07–2.38, p = 0.024) and a previous diagnosis of Renal disease (OR 2.43, 95% CI 1.50–3.92, p-value <0.001) remain statistically significant for the development of AKI in association with COVID-19 after adjusting for variables. Table 3.

### Survival analysis

Overall, 31.6% (n = 326) patients died following a diagnosis and admission for COVID-19. Overall mortality was significantly higher in the AKI group with 52.4% (n = 110) compared with 26.3% (n = 216) in the non-AKI group (p-value <0.001). Table 2.

Patients with AKI were more likely to be admitted to critical care (34.8%, n = 73 vs 11.2%, n = 92; p-value <0.001) and more likely to be ventilated once there (86.3%, n = 63 vs 66.3%, n = 61; p-value 0.006). They were also more likely to die, both in the critical care unit (52.1%, n = 38 vs 22.8%, n = 21, p-value <0.001) and overall (54.8%, n = 40 vs 25.0%, n = 23, p-value <0.001).

**Fig 1:**
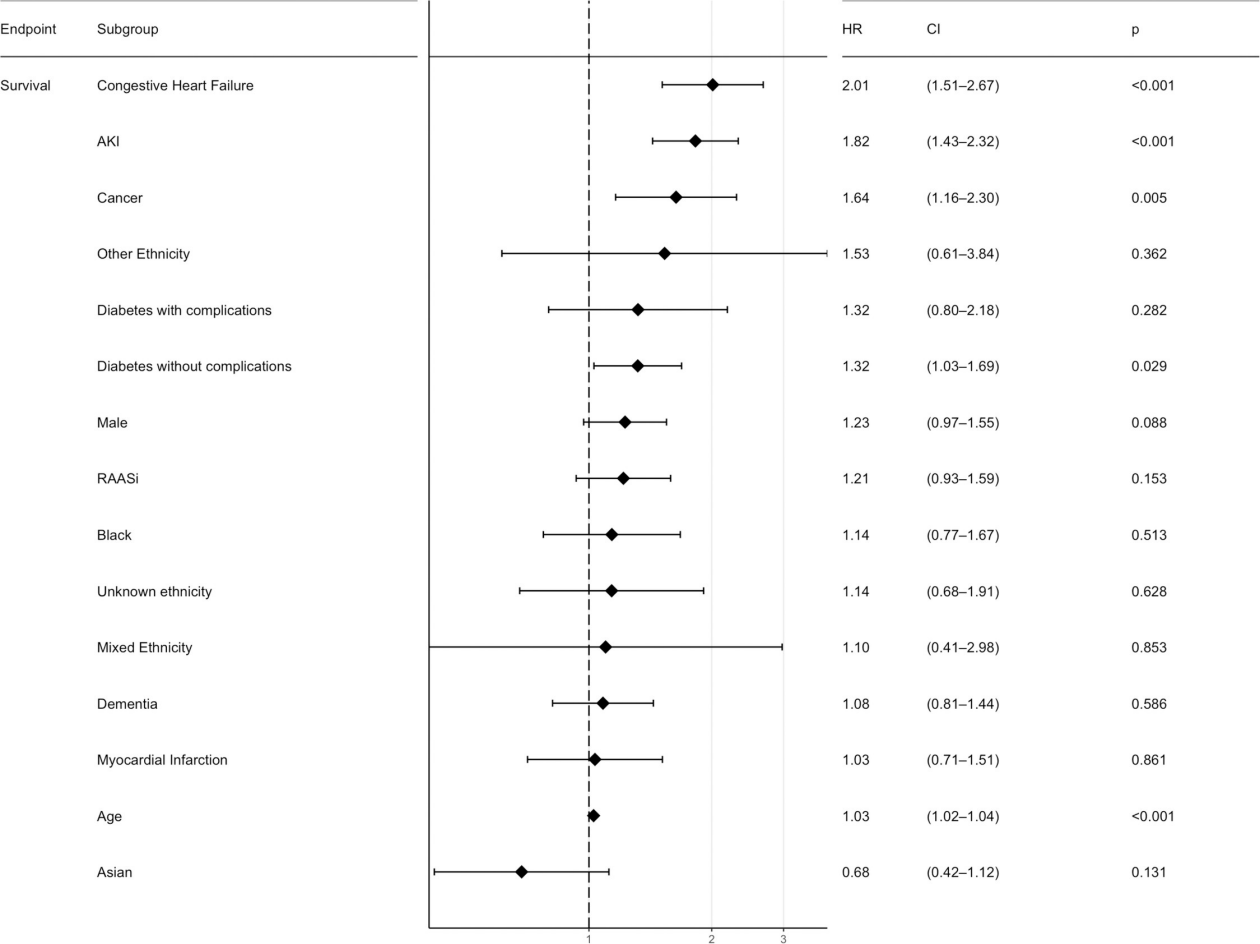
Multivariable analysis forest plot for overall survival from diagnosis with COVID-19. HR—Hazard Ratio; CI—95% Confidence Interval; AKI–Acute Kidney Injury; RAASi–Renin-angiotensin-aldosterone-system inhibitors; IMD–Index of multiple deprivation; CCI–Charlson Comorbidity index; COPD–Chronic Obstructive Pulmonary Disease.

**Fig 2:**
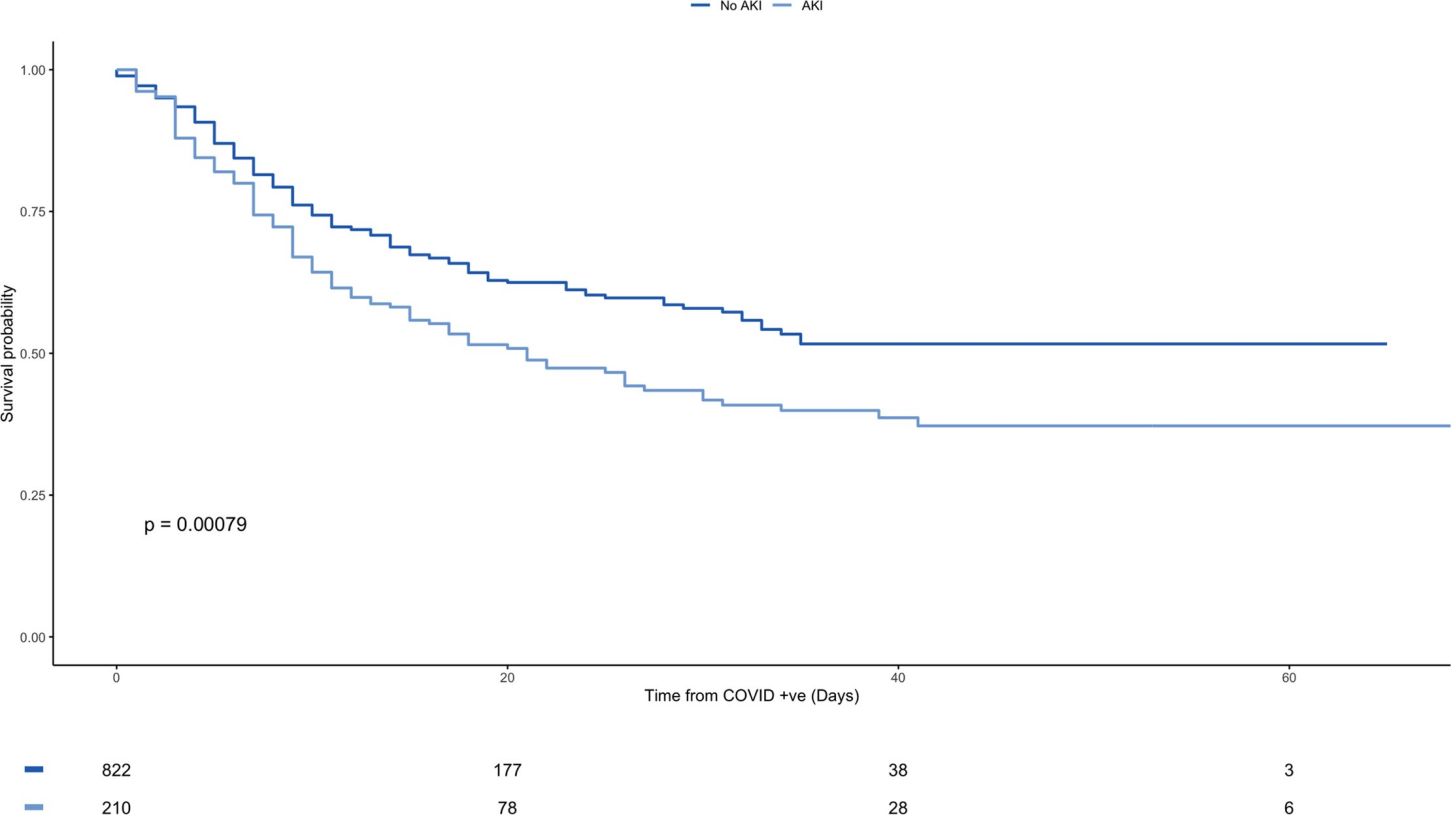
Kaplan-Meier plot for overall survival from diagnosis of COVID-19 stratified by AKI vs non-AKI.

For the whole study population, AKI is an independent risk factor for mortality with an HR 1.48 (CI 1.18–1.87, p-value <0.001) and it remains significant after adjusting for multiple variables with a HR 1.82 (CI 1.43–2.32, p-value <0.001). Figs 1 and 2, and S1 Table.

**Fig 3:**
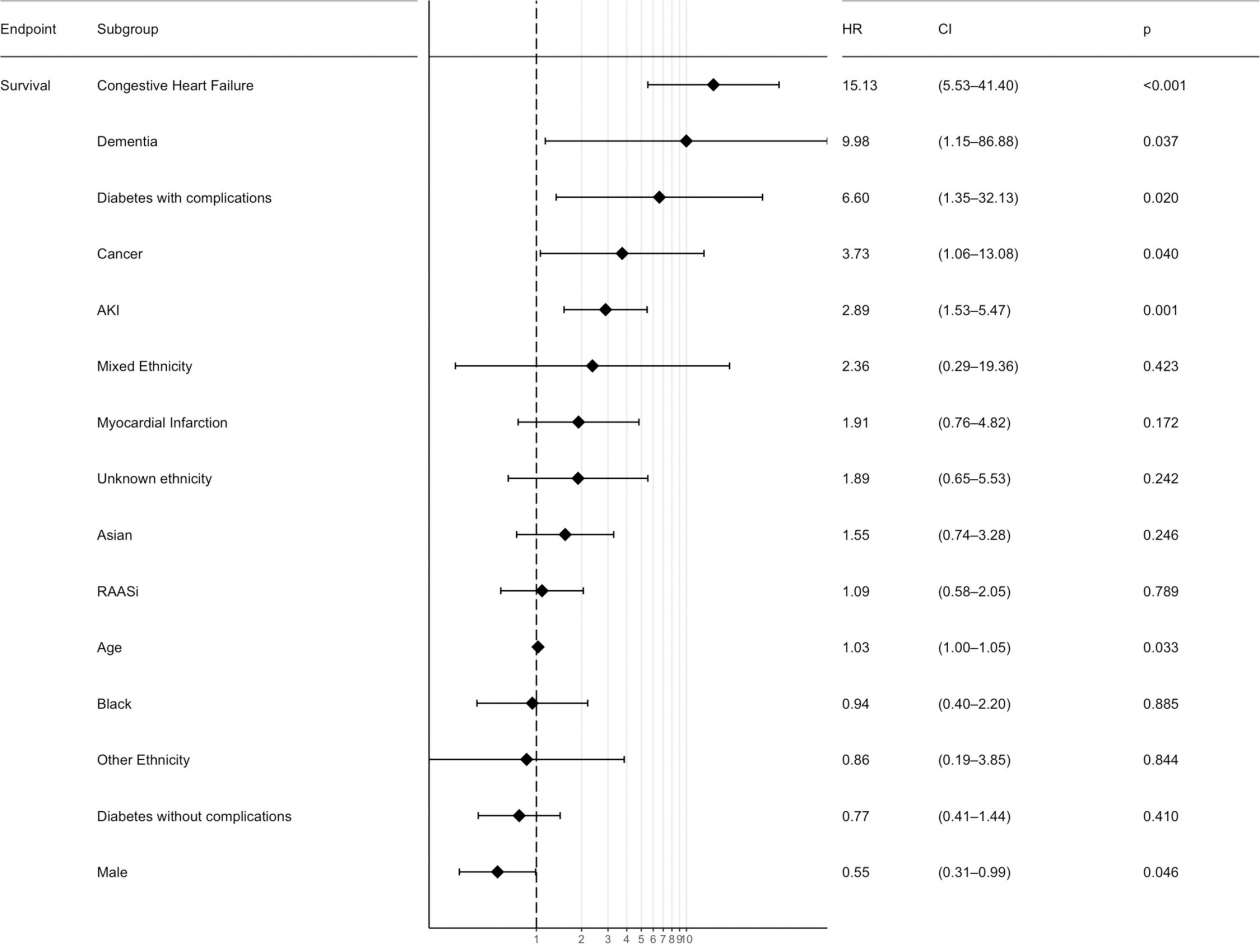
Multivariable analysis forest plot for survival following admission to critical care. HR—Hazard Ratio; CI—95% Confidence Interval; AKI–Acute Kidney Injury; RAASi–Renin-angiotensin-aldosterone-system inhibitors; IMD–Index of multiple deprivation; CCI–Charlson Comorbidity index; COPD–Chronic Obstructive Pulmonary Disease. Model based on clinically relevant parameters and with adequate number of events.

**Fig 4:**
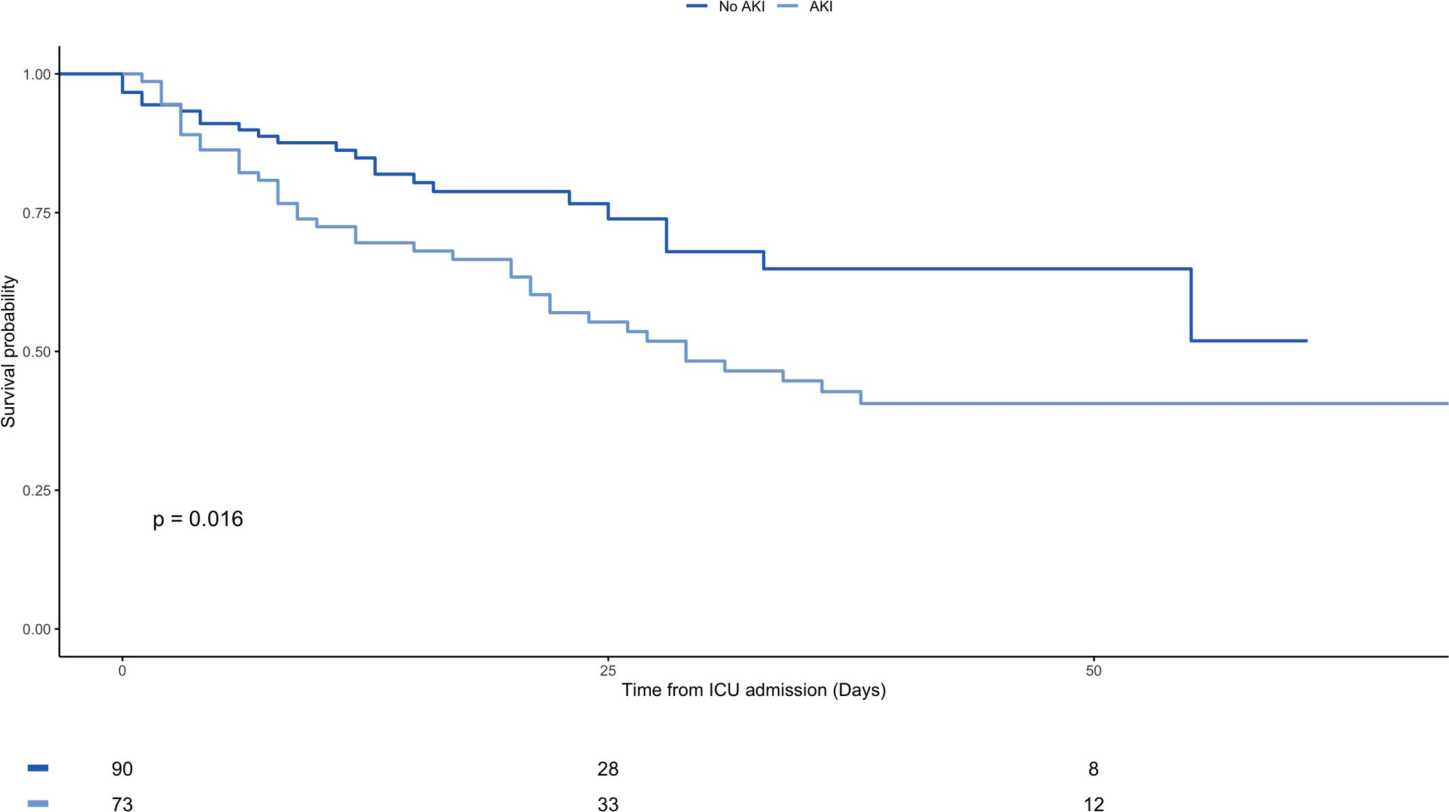
Kaplan-Meier plot for overall survival from admission to critical care stratified by AKI vs non-AKI.

For patients admitted to critical care, AKI is a statistically significant risk factor for death with a HR 2.05 (CI 1.22–3.42, p-value 0.006) and remains statistically significant at multivariable regression analysis with a HR 2.89 (CI 1.53–5.47, p-value 0.001). Figs 3 and 4, and S1 Table.

In the group of 100 AKI patients who survived, 82% (n = 82) of the patients recovered their kidney function, and only two patients remained dialysis dependent. Recovery was based on a creatinine level up to 50% above their baseline [29]. The mean number of AKI days (time to recovery) in those who survived was 15.2 (SD 10.5) with a median of 13 days (IQR 6–24).

There was no difference in overall mortality from either the initial stage of AKI or the peak AKI stage; HR 0.96, 95% CI 0.76–1.20, p-value 0.707 and HR 0.91, 95% CI 0.74–1.13, p-value 0.406 respectively. S1 Table and S1 and S2 Figs.

## Discussion

Our study found a high incidence of AKI in patients hospitalised with COVID-19 infection (20.3%), with the mortality in patients with AKI practically double that of patients without AKI (52.4% versus 26.3%). When the current COVID-19 pandemic emerged, initial reports suggested a respiratory limited condition with little multi-organ involvement. As the disease has progressed around the world the picture is evolving, and it is now evident that the disease results in extra-respiratory organ involvement, with significant prothrombotic disorders, neurological problems and cytokine storm patterns, as well as AKI, and all of these can have a dramatic impact on patient outcomes [10, 14, 16, 30–32].

We have analysed the outcomes of all patients admitted to a large teaching hospital in a diverse city in the UK. The patient cohort, although majority white, has a significant proportion of minority ethnic groups and a diverse cross section of social deprivation making it an ideal environment to study the disease across a range of demographics. ICNARC data showed greater than half of our ICU admissions came from social deprivation index scale 4 and 5 [33].

Fortunately, the vast majority of patients who contract COVID-19 appear to have a minor illness with little or no sequelae. Despite this, due to the vast numbers worldwide and its seeming predisposition in older, more comorbid patient groups, this has resulted in a significant cohort requiring hospitalisation which is placing a high burden on healthcare systems worldwide [34].

Many previous studies, including from our trust [20], have demonstrated AKI is a common complication among hospitalised patients but so far there are only a few studies among COVID 19 patients, and there has been a huge variation in the AKI incidence ranging from 0.5% to 36.6% [16]. Our study found AKI in 20.3%(n = 210) which was in line with the NY population.

In those hospitalised, the disease has a high mortality and morbidity as in our cohort of more than one thousand patients. Of the 1032 patients, 31.6% died following the diagnosis of COVID-19, compared to 14% deaths in all sectors across the UK as of 31st May 2020.

What is apparent from our study is the dramatic effect having an acute kidney injury has on the patients’ disease progression. In almost every metric studied, AKI results in significantly worse outcomes. Patients with AKI in association with COVID-19 are more likely to die (52.4% vs 26.3%) and more likely to be admitted to ICU (34.8% vs 11.2%). Once in ICU they were more likely to die in ICU (52.1% vs 22.8%), to die at any point following admission (54.8% vs 25.0%) and to require mechanical ventilation (86.3% vs 66.3%). Even adjusting for all other parameters, AKI remains a significant factor of overall mortality (HR 1.82, 95% CI 1.43–2.32, p-value <0.001) and following admission to ICU (HR 2.89, 95% CI 1.53–5.47, p-value 0.001). The mortality rates of ICU patients with AKI and COVID 19 are similar to those found in other European centres [18, 35].

Conversely, overall mortality in our cohort with AKI (52.4%) is higher than in that described by Hirsch et al. in New York City (36.6%) and in Wuhan, China (33.7% in patients with raised baseline creatinine) [10, 16]. Whilst a retrospective study provides limitations in fully explaining such a difference, it should be noted that a significant number of patients diagnosed with COVID-19 in the Manchester area were not admitted to the hospital, with the use of virtual wards to provide support for those with a milder disease course [22] (S1 File). The hospital is a tertiary hospital with a renal department, and so some patients were specifically transferred in from elsewhere for renal support. As such, the likely higher acuity of admitted patients could partly account for the higher mortality rate, as only those with a more severe phenotype were hospitalised. Certainly, there were more patients in our cohort who required escalation to critical care (16.0%) compared to both the Wuhan and NY study (10.4% and 9.7% respectively) [10, 16]. There has been much discussion about ethnicity as a risk factor, but this appears not to be the sole driver, as there were more White patients in the AKI group, compared to the non-AKI group in the NY cohort, along with a similar proportion of Black patients in both groups, which is the opposite to what we found in our study [16]. The differences are also not explained fully by diabetes, which was seen in only 14.3% of patients in the Wuhan cohort, but 33% in the NY population and both having similar mortality rates [10, 16].

There were a number of independent baseline characteristic risk factors associated with the development of AKI such as being male or black and being on RAAS inhibition similar to findings by Hirsch et al. (63.7% male and 20.8% black) but they did not find these drugs were related to greater AKI risk. Patients also had a higher serum sodium and haemoglobin, higher urea and creatinine, and a higher CRP, along with lower blood oxygen saturations and temperature. Once adjusted for all parameters, being male, having a higher baseline serum sodium and CRP as well as a previous diagnosis of Renal disease were all still significantly associated with AKI but RAAS inhibition was not.

Despite the poorer outlook for patients with AKI in the context of COVID-19 in regards to mortality, it seems if a patient survives they do appear to have a much higher chance of recovering their renal function (82%) compared to the 57% recovery in a study by Chan et al. [36]. This may be in part due to the unique nature of COVID-19 but also likely to be related to prior established robust early AKI interventions previously described within our hospitals [37]. Survival during and following ICU admission gives further insight into the apparent differences in COVID-19 compared to other severe respiratory conditions. In the AKI population, 38 patients died during their ICU stay with only two of the patients dying following discharge from critical care. This was similar in the non-AKI group where 21 patients died during their ICU stay, with only a further two dying on discharge. This would suggest that if a patient with COVID-19 survives their admission to ICU, they have a good chance of survival overall.

Similar to previous reports, the majority of our patients were found to have AKI on admission or within a day of diagnosis [16], with few developing it late in their disease course. Unlike in previous work on AKI associated with multiple other conditions, the stage of AKI (both the initial and peak) had no bearing on the mortality, it appears that just having AKI at all is a risk factor for worse outcomes.

Given the novel nature of the disease, the exact pathophysiology of AKI in COVID-19 has not been elicited fully as yet. It is, however, highly likely to be a multifactorial process involving any combination of, and not limited to, sepsis, nephrotoxins, hypovolaemia and hypotension, along with a background of renal disease. This is along with emerging data for more direct renal damage from endothelial injury, thrombotic events and glomerulopathy [11–13, 38–42]. Certainly, in our cohort, patients in the AKI group had a higher peak serum sodium at diagnosis, which may indicate a degree of hypovolaemia. RAASi was found significantly more in the AKI group than in the non-AKI group, although there was no difference in blood pressure at the time of diagnosis.

There are a number of limitations associated with this study in that it is a retrospective study with all the inherent bias associated with this. It has not been possible to determine the cause of death for patients aside from that patients died whilst being treated for COVID-19. It has also not been possible to say that AKI in of itself was a direct cause of mortality. It may be that the existence of AKI in a patient with COVID-19 is a surrogate marker for the severity of disease, as is already accepted in other diseases such as sepsis. The fact that baseline biochemistry such as CRP, and vital signs such as temperature, respiration rate and oxygen saturations were worse in patients with AKI does add some credence to this. Renal recovery was assessed based on serum creatinine. In the cohort of patients showing serological renal recovery there may be a subset who have still taken a significant “renal hit” as lower creatinine levels occur in patients recovering from an acute and/or prolonged illness. This is in part due to a loss of muscle bulk and poor dietary intake. We could not assess this which may then overestimate the true renal function. Given this was a retrospective study, there were also multiple parameters that were unable to be studied due to the amount of missing data, including weight and body mass index, along with biochemistry such as Ferritin, Troponin T, Lactate Dehydrogenase, D-Dimer, Procalcitonin, Creatinine Kinase, pro-B-type natriuretic peptide, and urinalysis.

## Conclusion

Despite the limitations, this study, across a diverse large population in the UK, shows that the development of AKI, no matter how severe, is associated with dramatically worse outcomes for patients in terms of mortality and the requirement of critical care admission and organ support. Patients presenting with AKI and those at risk of AKI, with suspected COVID-19 should be closely monitored given the risk of deterioration and death. With the findings of this study future strategies to deal with COVID-19 should include systems and processes for rapid detection, prevention and proactively management acute kidney injury. Future prospectively designed studies on COVID-19 should consider management strategies that minimise the potential for AKI.

## Supporting information

S1 Table Univariable analysis for overall survival and survival following admission to critical care. AKI–Acute Kidney Injury; BAME–Black, Asian and Minority Ethnic; RAASi–Renin-angiotensin-aldosterone-system inhibitors; IMD–Index of multiple deprivation; CCI–Charlson Comorbidity index; COPD–Chronic Obstructive Pulmonary Disease.
(DOCX)

S1 Fig Kaplan-Meier plot for overall survival stratified by initial AKI stage. (TIF)

S2 Fig Kaplan-Meier plot for overall survival stratified by peak AKI stage. (TIF)

S1 File Manchester University NHS Foundation Trust COVID-19 virtual ward pathway. (PDF)
