# Supplementary material for: Characteristics and outcomes of hospitalised patients with acute kidney injury and COVID-19
Source: PLoS One. 2020 Nov 3;15(11):e0241544. doi: 10.1371/journal.pone.0241544 (PMC7608889; doi:10.1371/journal.pone.0241544)
Supplement: S1 Table — AKI–Acute Kidney Injury; BAME–Black, Asian and Minority Ethnic; RAASi–Renin-angiotensin-aldosterone-system inhibitors; IMD–Index of multiple deprivation; CCI–Charlson Comorbidity index; COPD–Chronic Obstructive Pulmonary Disease. (DOCX) [file pone.0241544.s001.docx]

| Survival | Overall survival | | Following admission to critical care | |
| --- | --- | --- | --- | --- |
|  | HR (95% CI) | p-value | HR (95% CI) | p-value |
| AKI | 1.48 (1.18-1.87) | 0.001 | 2.05 (1.22-3.42) | 0.006 |
| Age | 1.03 (1.02-1.04) | 0.001 | 1.04 (1.02-1.06) | <0.001 |
| Sex - Male | 1.18 (0.94-1.47) | 0.150 | 0.74 (0.44-1.23) | 0.241 |
| Ethnicity - White | Reference | | | |
| Ethnicity - Asian | 0.59 (0.38-0.91) | 0.018 | 0.92 (0.46-1.82) | 0.806 |
| Ethnicity - Black | 1.13 (0.79-1.63) | 0.502 | 1.00 (0.49-2.05) | 0.989 |
| Ethnicity - Mixed | 0.76 (0.28-2.04) | 0.581 | 0.39 (0.05-2.83) | 0.348 |
| Ethnicity - Other | 0.67 (0.28-1.63) | 0.379 | 1.41 (0.43-4.61) | 0.573 |
| Ethnicity- Unknown | 0.86 (0.53-1.38) | 0.528 | 1.10 (0.46-2.64) | 0.826 |
| BAME - White | Reference | | | |
| BAME - BAME | 0.81 (0.61-1.07) | 0.132 | 0.94 (0.56-1.58) | 0.812 |
| BAME - Unknown | 0.86 (0.53-1.38) | 0.528 | 1.10 (0.46-2.65) | 0.823 |
| RAASi | 1.44 (1.13-1.85) | 0.004 | 2.18 (1.30-3.64) | 0.003 |
| IMD - Decile | 0.98 (0.95-1.02) | 0.332 | 0.91 (0.83-1.00) | 0.045 |
| CCI Score | 1.27 (1.17-1.37) | 0.001 | 1.69 (1.28-2.24) | <0.001 |
| Myocardial Infarction | 1.44 (1.01-2.06) | 0.044 | 4.46 (1.87-10.66) | 0.001 |
| Congestive Heart Failure | 2.32 (1.78-3.02) | 0.001 | 5.97 (2.80-12.74) | <0.001 |
| Peripheral Vascular Disease | 1.06 (0.72-1.58) | 0.757 | 2.38 (0.94-6.00) | 0.066 |
| Cerebrovascular Disease | 1.18 (0.75-1.83) | 0.475 | 2.27 (0.82-6.31) | 0.115 |
| Dementia | 1.43 (1.11-1.85) | 0.006 | 3.60 (0.49-26.33) | 0.207 |
| COPD | 1.05 (0.83-1.34) | 0.670 | 0.83 (0.46-1.48) | 0.525 |
| Rheumatoid Disease | 1.21 (0.63-2.36) | 0.566 | NA (NA-NA) | NA |
| Peptic Ulcer Disease | 0.27 (0.04-1.95) | 0.196 | 0.00 (0.00-Inf) | 0.996 |
| Mild Liver Disease | 0.54 (0.22-1.30) | 0.168 | 0.00 (0.00-Inf) | 0.996 |
| Diabetes without complications | 1.32 (1.04-1.68) | 0.023 | 1.10 (0.63-1.90) | 0.744 |
| Diabetes with complications | 1.68 (1.04-2.70) | 0.033 | 2.71 (0.65-11.21) | 0.169 |
| Hemi or Paraplegia | 0.54 (0.20-1.44) | 0.215 | 0.00 (0.00-Inf) | 0.996 |
| Renal Disease | 1.56 (1.20-2.02) | 0.001 | 3.52 (1.90-6.51) | <0.001 |
| Mod/Severe Liver Disease | 1.73 (0.71-4.19) | 0.224 | 1.31 (0.18-9.47) | 0.791 |
| Cancer | 1.94 (1.39-2.70) | 0.001 | 4.25 (1.30-13.87) | 0.016 |
| Metastatic Cancer | 2.23 (1.28-3.89) | 0.005 | NA (NA-NA) | NA |
| AIDS | 5.62 (0.79-40.20) | 0.085 | NA (NA-NA) | NA |
| Initial AKI stage | 0.96 (0.76-1.20) | 0.707 | 1.23 (0.84-1.79) | 0.282 |
| Peak AKI stage | 0.91 (0.74-1.13) | 0.406 | 1.63 (1.00-2.67) | 0.051 |

Table 4 – Univariable analysis for overall survival and survival following admission to critical care. *AKI – Acute Kidney Injury; BAME – Black, Asian and Minority Ethnic; RAASi – Renin-angiotensin-aldosterone-system inhibitors; IMD – Index of multiple deprivation; CCI – Charlson Comorbidity index; COPD – Chronic Obstructive Pulmonary Disease.*
