# Supplementary figures and images for: Characteristics and outcomes of hospitalised patients with acute kidney injury and COVID-19
Source: PLoS One. 2020 Nov 3;15(11):e0241544. doi: 10.1371/journal.pone.0241544 (PMC7608889; doi:10.1371/journal.pone.0241544)

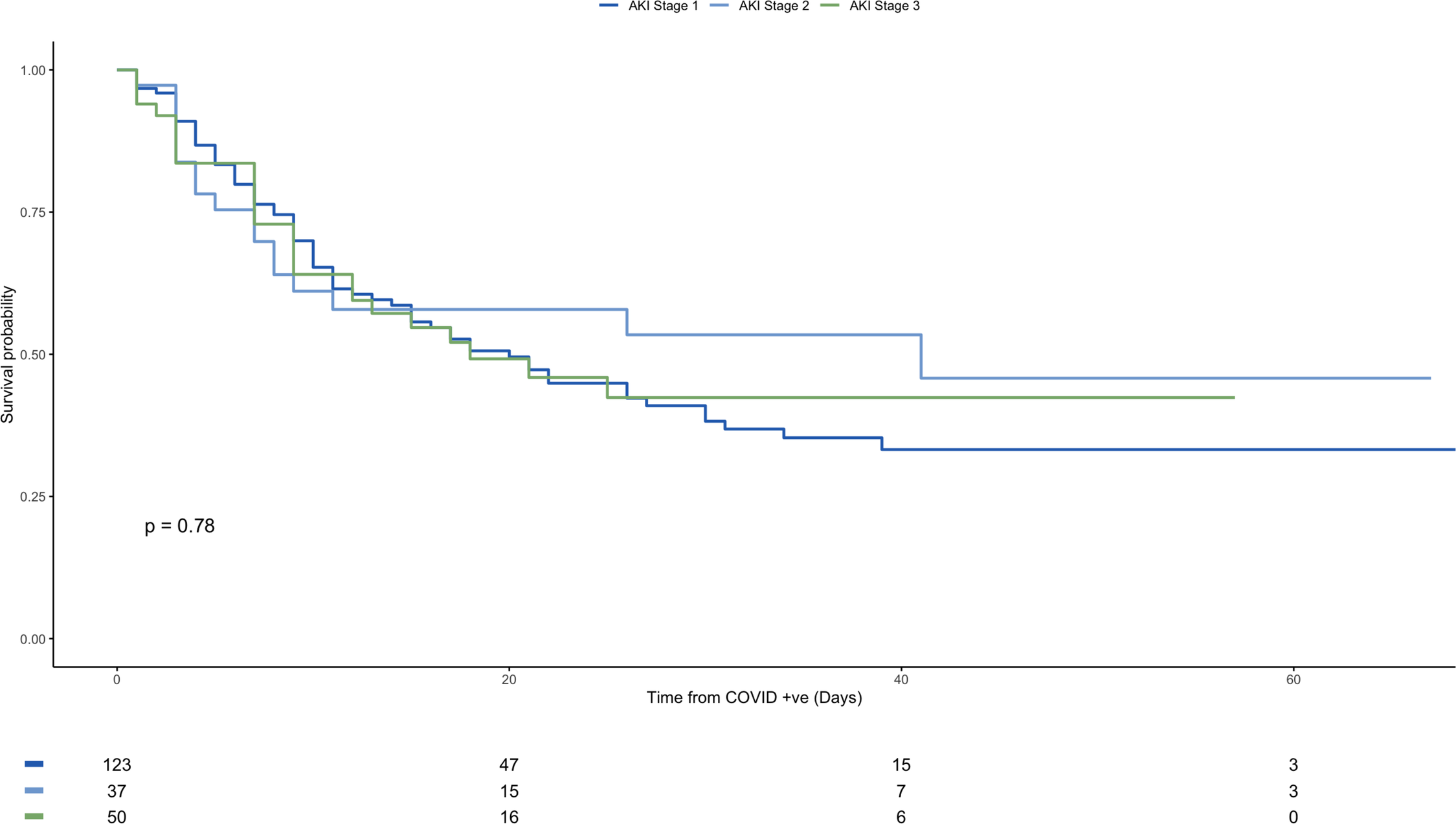

Supplement: S1 Fig — (TIF) [file pone.0241544.s002.tif]

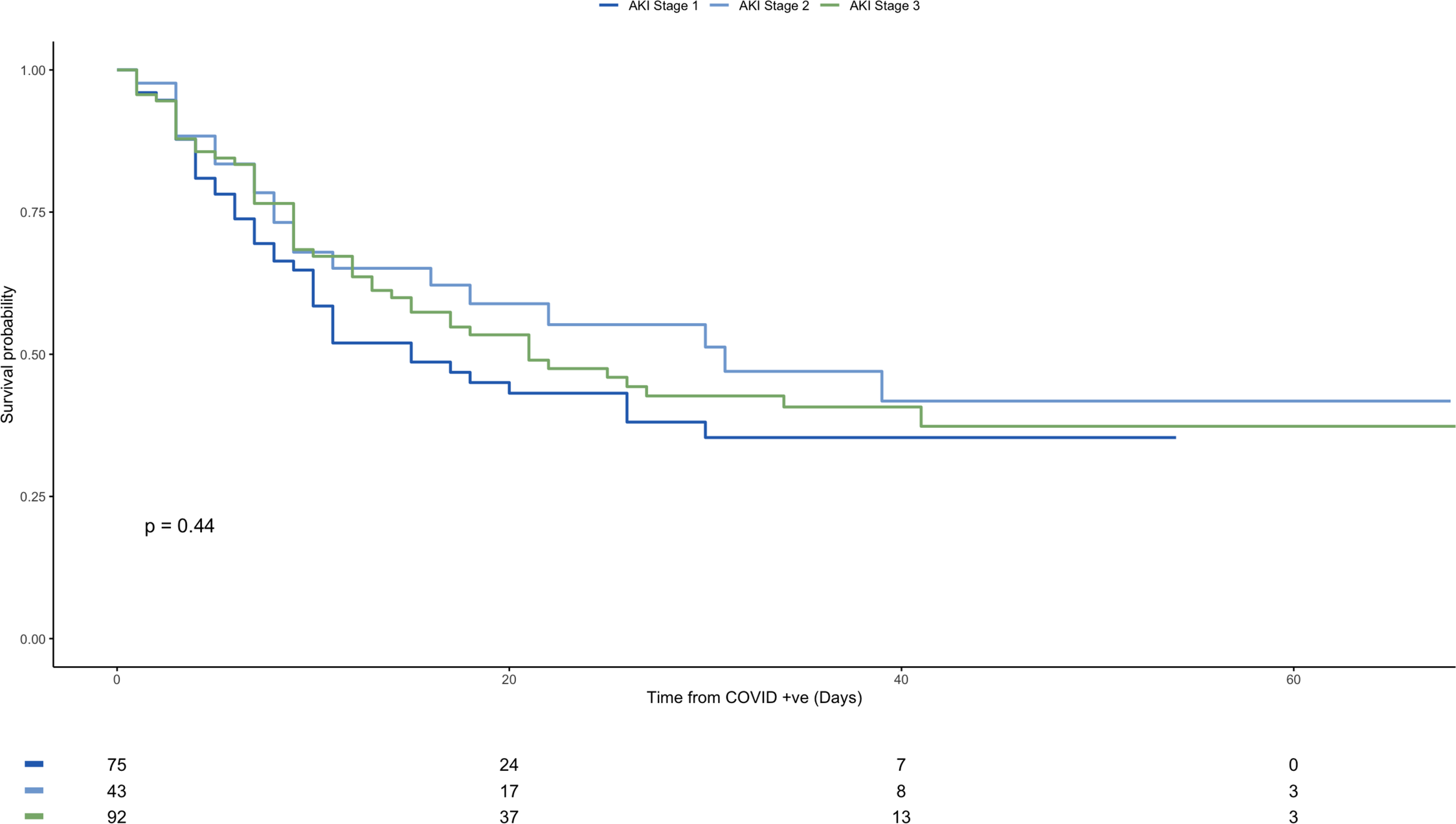

Supplement: S2 Fig — (TIF) [file pone.0241544.s003.tif]
