# Supplementary material for: Characteristics and outcomes of hospitalised patients with acute kidney injury and COVID-19
Source: PLoS One. 2020 Nov 3;15(11):e0241544. doi: 10.1371/journal.pone.0241544 (PMC7608889; doi:10.1371/journal.pone.0241544)
Supplement: S1 File — (PDF) [file pone.0241544.s004.pdf]

NHS
Manchester University
NHS Foundation Trust

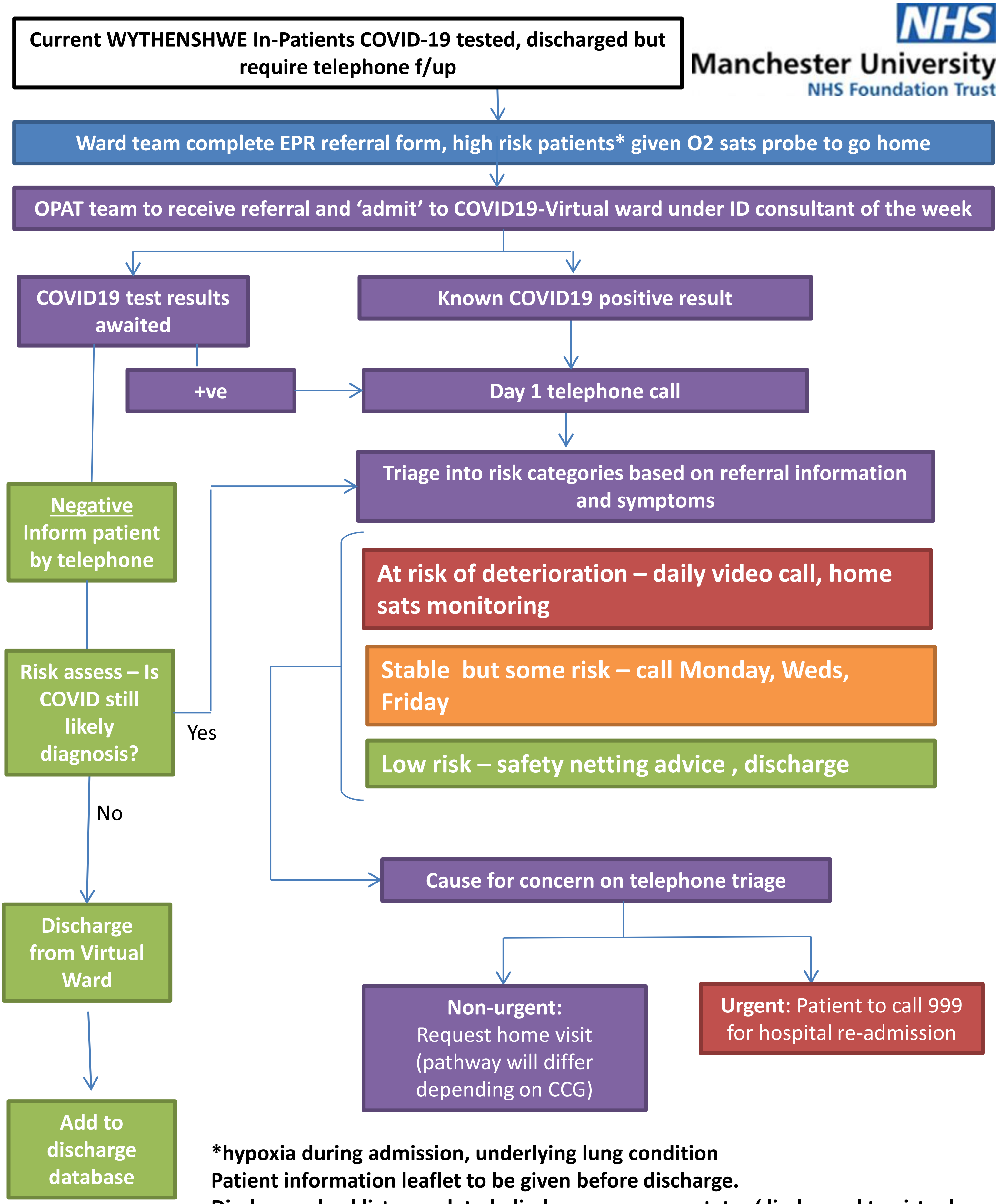

***hypoxia during admission, underlying lung condition**
Patient information leaflet to be given before discharge.
Discharge checklist completed, discharge summary states 'discharged to virtual ward'

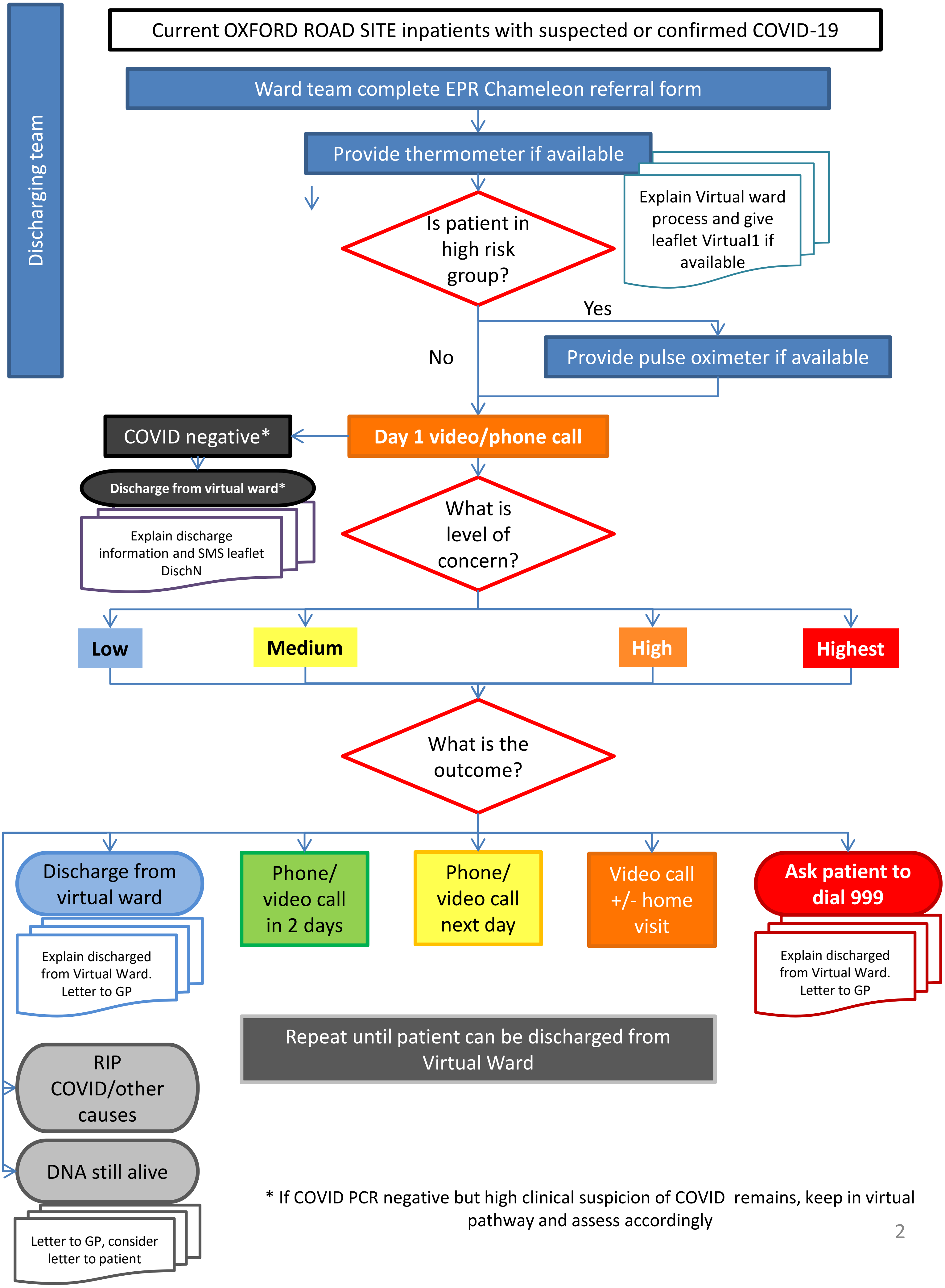

* If COVID PCR negative but high clinical suspicion of COVID remains, keep in virtual pathway and assess accordingly

Wythenshawe Hospital Team – 8am – 5pm (7 days)

Name	Title	Role	Backup in case of sickness
Binita Kane<br>Riina Richardson<br>Giorgio Calisti<br>Rohit Bazaz	Consultant	Oversight of service<br>Specialist advice	Cross-cover for sickness<br>Ashley Woodcock<br>Caroline Baxter
Helen Bradbury<br><br>Carole Toner	Administrator (WTE B2)<br><br>HCS B3	Assisting in Virtual Ward administration	All OPAT members trained in these roles
Liz Wilson	Band 8 lead	Nursing lead for service<br><br>Oversight of VW Processes and nursing team	Consultants/OPAT nurses
Vicky Whitehead<br>Lynn Connor<br>Ben Freeman<br>Raquel Gonzales	Band 6 nurses	Telephone follow-up of patients	Cross-cover between team<br><br>Other NWLC staff

Oxford Road Team – 8.30am – 4.30pm (7 days)

Name	Title	Role	Backup in case of sickness
Tom Gorsuch<br>Waseem Khan<br>Seema Brij	Consultant	Oversight of service<br>Specialist advice	Respiratory Consultants
Sundilla Rafiq<br>Katie Birch	Medical secretary / assistant medical secretary	Assisting in Virtual Ward administration	Kathryn Birchenough
Shane O'Reilly	Band 8 lead	Clinical and managerial lead for service<br><br>Oversight of VW processes and nurse/physio team	Consultant / senior COPD team
COPD Team<br>Asthma Team	Band 6/7 nurses and AHPs	Telephone follow-up of patients<br>Home Visits as required	Respiratory Physiologists

Process for managing virtual ward

1) Day 1 patients coded ‘red’
    - Triage into traffic light system by telephone assessment following proforma
    - Call details recorded via a communications note on EPR
    - Tracking board updated

RED – daily phone call /video call using proforma questions
AMBER – call Monday, Wednesday, Friday
GREEN – safety netting advice, consider 1 further call or discharge.

2) Board round midday to discuss patients and any concerns.

3) Patients triggering ‘cause for concern’
- Establish which CCG their GP practice falls into
- If urgent ask patient to call 999, patient to inform NWAS of COVID status
- For non-urgent patients, request same day visit following guidance from individual CCG

4) Discharging patients
- Remove from tracking board and transfer to discharge list
- Generate GP letter via Winscribe / Medisec acc to template
- Advice: no need to self isolate if >14 days from onset of symptoms
- General safety netting advice, if need for ongoing clinical review – follow usual pathways (e.g. GP, community teams if know to them)
- No need to follow PHE guidance for high-risk patients as should have immunity

Telephone follow-up questions

Wythenshawe

Daily phone calls:

Q: How is your breathing today?

Q: Is it better, worse, no change from yesterday?

Q: Are you breathing harder or faster than usual when doing nothing at all?

Q: What could you do yesterday that you can't do today?

Q: What makes you breathless now that didn't make you breathless yesterday?

- **Dyspnoea or Tachypnoeia +/- hypoxia = ALERT**

Q: Are there any other symptoms causing you concern?

If so, document the concern and discuss on the daily boardround if symptom does not trigger immediate medical review or 999 transfer to hospital

Discuss self-isolation/social distancing.

Discuss isolation of household members.

Agree next steps i.e. when to expect the next phone call

Ensure the pt knows how & when to escalate (see PIL)
Any other questions?

Document via communication note on EPR

Oxford Road Site

Day 1 phone call

- Introduction
- Confirm ID
- Ask how they are feeling
- Provide COVID result if available (if negative, see below)
- Ask set questions
- Explain and SMS plan
- Remind isolation rules
- Offer to SMS Virtual 1 if not received
- Contact phone numbers GP/111/999

Daily questions

- How is your breathing today?
  - Is it better, the same or worse than yesterday?
  - Are you more breathless than usual when you are not doing anything?
- (If worse) Is there anything you could do yesterday which you can't today?
- What are the readings on your oxygen probe?
  - Heart rate (HR), oxygen saturation (SpO2), temp
- Are you coughing? Is this better/the same/worse than yesterday?
- Are you bringing up phlegm or blood? Is this better/the same/worse than yesterday?
- Are there any other symptoms you are concerned about?

Subsequent phone calls

- Introduction
- Confirm ID
- Ask how they are feeling
- Provide COVID result when available (if negative, see below)
- Ask set questions
- Explain and SMS plan
- Remind isolation rules
- Offer to SMS Virtual 1 if not received
- Contact phone numbers GP/111/999

Home visit - Manchester

Central Manchester

Contact Central Community Respiratory Team

Telephone: 0161 276 6035

South Manchester

Discuss with Dr Kane or Community Respiratory Team (CRT) whether patient is appropriate for CRT review

CRT: 01612915965 or page via switchboard

If not appropriate, discuss with COVID GP home review service (details awaited)

CCG/Community leads for escalation of issues

Murugeshan Raja (CCG lead)

murugesan.raja@nhs.net

Mobile: 07963876942

Doug Jeffrey (CD for Community services)

doug.jeffrey@nhs.net

Mobile: 07590 670755

Home visits - Trafford

Manchester University NHS Foundation Trust

Telephone: **0161 975 4741** to give details of patient
Operational Hours: 0800 – 1600hrs

Email: tspoa1@nhs.net
In subject: type 'FAO Trafford Matrons' to confirm details in writing

Trafford CCG/Community contacts for escalation of issues
Brooks Kenny (Commissioner)
brookskenny@nhs.net
07971483828

Richard Spearing (Trafford LCO)
richard.spearing@nhs.net
07920581980

Lesley Lyons (Lead for Trafford Matrons)
llyons@nhs.net
07415242389

Trafford Matrons acute case load is full – a/w details of COVID hot hubs and home visiting service

Home visits - Stockport

The Crisis Response Team can be contacted on: **0161 476 9665**
Operational hours: 8am – 10pm, 7 days a week
Multi-disciplinary team

CCG/Community Contacts for escalation of issues

nicole.alkemade@nhs.net Commissioning lead
Mobile: 07795267081

Jayne Etches
Professional Service Lead |Crisis Response Team |1st Floor |Kingsgate House|Wellington Road South |Stockport|SK4 1LW
07810-816214
Jayne.etches@nhs.net

Lisa Lainton
Head of Borough Wide Services
Integrated Care Business Group
Currently based in Estates Dept.
Stockport
SK2 4JT
temp No. 07760666370
Days worked. Mon, Tues, Thurs, Friday.
Please note new email lisajane.lainton@nhs.net

Manchester University NHS Foundation Trust

PHE guidance on PPE and isolation

- For the confirmed COVID cases that are discharged from hospital, self-isolation can be lifted 7 days after symptom onset according to PHE guidelines updated on 18/03/20 link:https://www.gov.uk/government/publications/covid-19-stay-at-home-guidance/stay-at-home-guidance-for-households-with-possible-coronavirus-covid-19-infection
- Household contacts need to remain in self-isolation for longer - 14 days from onset of symptoms in the household index case.

- **After 7 days from symptoms onset, staff visiting the confirmed case at home do not need to wear PPE**
- If there are other people in the same household that are symptomatic and have been unwell for less than 7 days, the visiting staff should avoid any contact with them.

NHS
Manchester University
NHS Foundation Trust

Team contact details

South Manchester Virtual Ward team:

Call OPAT on 0161 291 4207 (staff only)

Patients will be provided with a separate number manned by NWLC nurses.

For Senior Medical Advice

Call Wythenshawe switchboard: 01619987070 and ask for the Infectious Diseases (ID) Consultant on-call

** further details of Resp consultant advice and guidance to be provided**

For logistic issues regarding the virtual ward or community pathways

Wythenshawe: call switchboard 01619987070 and ask for Dr Kane on her mobile phone

Central Manchester Virtual Ward team:

Call 0161 276 4379 or 0161 276 6035

For Senior Medical Advice

Speak to COVID admissions unit Consultant

For logistic issues regarding the virtual ward or community pathways

MRI: call switchboard 0161 276 6035 ask to speak to Shane O’Reilly or senior member of COPD team if not available

Workforce requirements (per site)

Virtual ward numbers	Average daily phone calls	Average weekly phone calls 7/7	Total time required (hours)	Spec Nurse (WTE)	Consultant daily hours	Consultant weekly hours (7/7)	Consultant PAs
20	10	70	23.3	0.6	0.5	3.5	0.9
40	20	140	46.7	1.2	1.0	7	1.8
60	30	210	70.0	1.8	1.5	10.5	2.6
80	40	280	93.3	2.3	3.0	21	5.3
100	50	350	116.7	2.9	2.5	17.5	4.4
120	60	420	140.0	3.5	5.0	35	8.8
140	70	490	163.3	4.1	3.5	24.5	6.1

*based on each phone call plus administration requiring 20 minutes

Each time numbers increase by 20, a staffing review will be triggered. Staff will be added to the virtual ward team from a bank of clinical staff who have been redeployed from the front line.

This modelling relates only to phone calls and does not take into account the number of visits that may be required. This is as yet an unknown quantity but will be kept under review as the model progresses. As a general rule one member of qualified staff will be able to undertake a maximum of 6 home visits in a day.

Performance Measures

- Number of daily referrals
- Number of readmissions within 7 days and 14 days.

Audit and publication

Additional data will be collected by clinical teams for anonymized rapid publication of service evaluation.

NHS
Manchester University
NHS Foundation Trust

Management of COVID results at Wythenshawe* – role of virtual ward

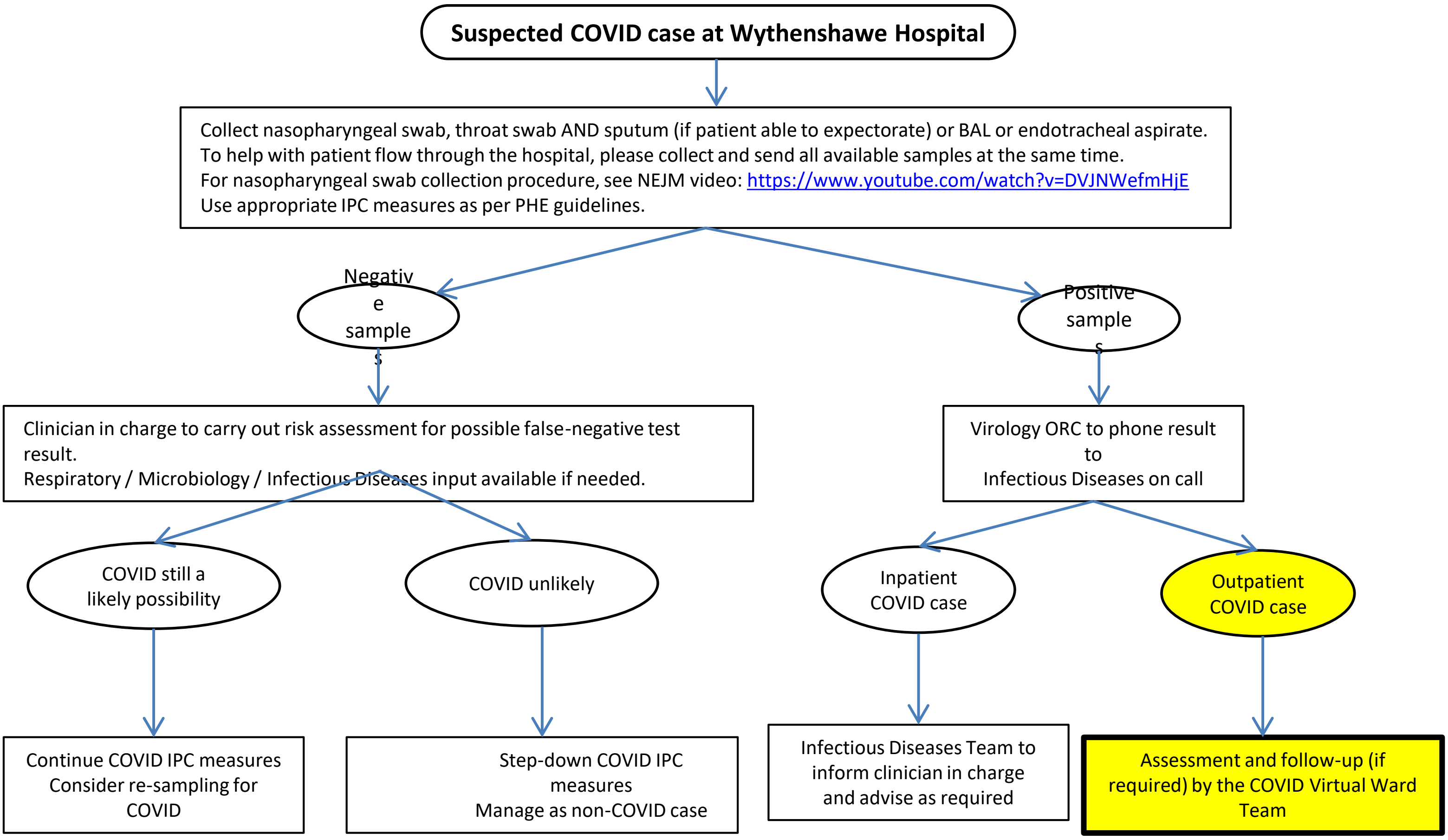

*ORC pathway TBC
